# Rare case report of infective endocarditis due to *Kocuria kristinae* in a patient with ventricular septal defect

**DOI:** 10.1099/acmi.0.000076

**Published:** 2019-11-01

**Authors:** Arif Maqsood Ali, Gule Raana Waseem, Shazia Arif

**Affiliations:** ^1^​ Department of Pathology and Blood Bank, Rawalpindi Institute of Cardiology, Rawalpindi, Pakistan; ^2^​ Rawalpindi Institute of Cardiology, Rawalpindi, Pakistan; ^3^​ AMC, Rawalpindi, Pakistan

**Keywords:** Endocarditis, Ventricular septal defect, Kocuria kristinae

## Abstract

**Background:**

Infective endocarditis (IE) is an uncommon but life-threatening infection. It is commonly associated with diseased or damaged valves. Patients with congenital heart disease are more prone to getting IE than the general population. The typical organisms that cause IE include *Staphylococcus *, *Coagulase-negative Staphylococcus*, *Streptococcus viridians* and *Enterococci*. However, the importance of rare micro-organisms like *Kocuria kristinae *should not be underestimated especially when isolated from multiple blood cultures in patients suspected of IE.

**Case presentation:**

We report a rare case of right-sided infective endocarditis due to *K. kristinae* in a young non-diabetic, non-addict female of low socioeconomic class who presented with undiagnosed fever for 1 year. She was investigated and treated for fever by several general practitioners without relief. Later on, she was diagnosed by a local cardiologist to have perimembranous ventricular septal defect with a small pulmonary valve vegetation. She was referred to a tertiary care cardiac hospital in Rawalpindi, Pakistan for further management. Transthoracic and transesophageal echocardiography confirmed IE secondary to preexisting congenital heart disease complicated with a small pulmonary vegetation. Her blood cultures yielded growth of *K. kristanae,* a rare micro-organism to cause IE. The patient responded to the antibiotic therapy.

**Conclusion:**

Clinicians should have a high index of suspicion for *K. kristanae* IE as a possible cause of a prolonged fever especially in the presence of congenital heart disease. Antibiotic susceptibility is required for adequate therapy.

## Introduction

Ventricular septal defect (VSD) being the commonest congenital heart disease (CHD) poses a high risk of infective endocarditis (IE) [1]. Being a part of oral and skin microbiota *Streptococcus viridans*, *Coagulase negative Staphylococci* are the commonest and *Kocuria kristinae* is a rare cause of IE [2, 3]. *K. kristinae* was previously known as *Micrococcus kristina* [4, 5]. It belongs to the family *Micrococcaceae *, suborder Micrococcineae, order Actinomycetales, class Actinobacteria [6]. Nineteen species of *Kocuria* species have been identified so far [7]. It includes Gram-positive, strictly aerobic, catalase-positive, coagulase-negative, non-haemolytic cocci. It frequently colonizes the skin, mucosa and oropharynx [6]*. K. kristinae* has recently gained attention because of its potential to cause disease in humans. It has been shown that *Kocuria rosae, K. kristinae and Leuconostic mesenteroides* are involved in the development of dental caries [8]. *K. kristinae* has been reported to cause infection in patients with central venous catheters, dialysis-associated peritonitis, acute cholecystitis and black tongue [9]. It is most commonly isolated from the blood cultures of infants, immunocompromised patients and patients with catheter-related bacteremia [4, 9].

The predisposing factors associated with infections related to *Kocuria* spp. include congenital deformities, long-term catheters, malignancies and patients with end-stage renal disease undergoing continuous ambulatory peritoneal dialysis [10]. Other underlying conditions associated with *Kocuria* infection include diabetes mellitus, tuberculosis, stem-cell transplant patients, patients suffering from gallstones, methylmalonic aciduria and pancreatic pseudocyst [7].

The prevalence of infection due to *Kocuria* spp. might be underestimated considering their close similarity to *Coagulase negative Staphylococci* [7]. Rare case reports of *K. kristinae* have been cited in the literature[9–11]. There are only five patients reported in the literature with IE caused by *K. kristinae *. Moreover, there are no antibiotic guidelines for the treatment of *K. kristinae* infection [12].

Here we report a rare case of a patient with a history of untreated perimembranous ventricular septal defect (PMVSD) with pulmonary valve vegetation who presented to us with a history of intermittent fever for the last year.

## Case report

A young non-diabetic, non-addict female of low socioeconomic class, with poor dental hygiene and carious teeth from Nowshera district of Khyber Pakhtunkhaw province of Pakistan in her late twenties presented with a history of intermittent fever for 1 year. The fever was higher than 38 °C and lasted for 2 to 3 h daily. There was an associated history of arthralgia. There were reddish petechial spots on her lower shin bones and non-tender macular lesions on her palms and soles of feet. There was no past history of any medical, surgical, gynecological or obstetric disease. She had two sons of 10 and 5 years of age. She did not have a family history of congenital heart disease, endocarditis, autoimmune disease, diabetes mellitus, hypertension, ischemic heart disease, tuberculosis or any other disease. She was investigated for fever. She had haemoglobin of 9.6 g dl^−1^ and an erythrocyte sedimentation rate (ESR) 78 mm at the end of first hour. There was microscopic haematuria and mild proteinuria in urine routine examination. Rheumatoid factor (RA), anti-cyclic citrullinated peptide (Anti-CCP) and typhidot were negative. Liver and renal function tests were normal. Her white blood cell (WBC) count was 14.3×10^9^ l^−1^ and complement reactive protein (CRP) 56 g dl^−1^. She was managed by local general physicians with symptomatic treatment with analgesics, antipyretics, broad spectrum oral and parenteral antibiotics including ampicillin, tetracycline, azithromycin, ciprofloxacin, linezolid, vancomycin and antimalarials. However, the patient did not improve.

**Fig. 1.**
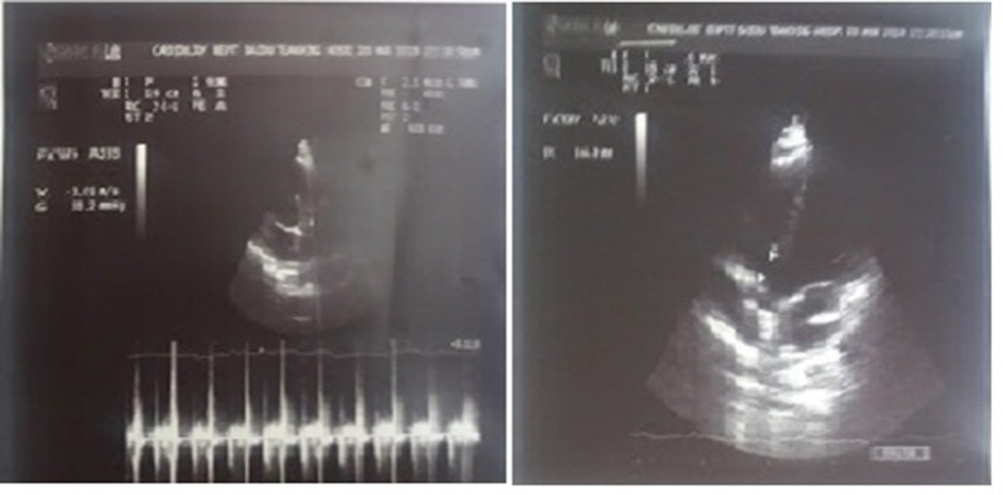
Transthoracic echocardiography showing a vegetation in the pulmonary valve and ventralseptal defect.

She consulted a local cardiologist who identified a moderate PMVSD and a small 7 mm vegetation attached to pulmonary valve on transthoracic echocardiography (Fig. 1). Blood culture was advised, which yielded *Coagulase-negative Staphylococcus*. She was referred to Rawalpindi Institute of Cardiology, Rawalpindi (RIC), which is a tertiary care hospital in the twin capital city of Rawalpindi/Islamabad, Pakistan for further management. Repeat transthoracic and transesophageal echocardiography at RIC confirmed PMVSD and a small pulmonary valve vegetation.

Three sets of blood cultures were taken by strict aseptic technique at 12 h interval. Then, 10 ml of venous blood drawn peripherally was inoculated into each blood culture bottle and incubated in Bact T/Alert 3-D bioMerieux, France. Intravenous (i.v) Ceftriaxone 1 g d^−1^, i.v vancomycin and i.v amikacin 500 mg twice daily were started empirically. All three blood cultures were flagged positive after 48 h. Gram stain of positive blood culture bottles showed Gram-positive cocci in pairs, tetrads and in small groups. Subcultures on Blood Agar and Chocolate Agar yielded small convex, smooth, creamy white, non-haemolytic colonies that were Gram-positive in pairs, tetrads, catalase-positive, coagulase-negative and DNAse-negative. These showed resistance to nitofurantoin but sensitivity to bacitracin.

**Fig. 2.**
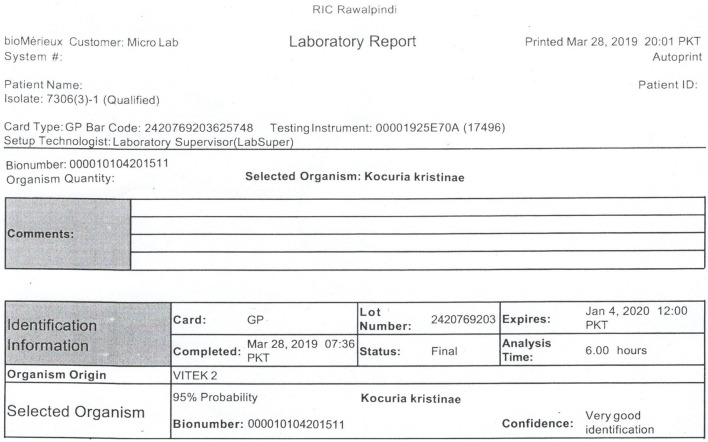
Identification of *K. kristinae* by Vitek-2.

**Fig. 3.**
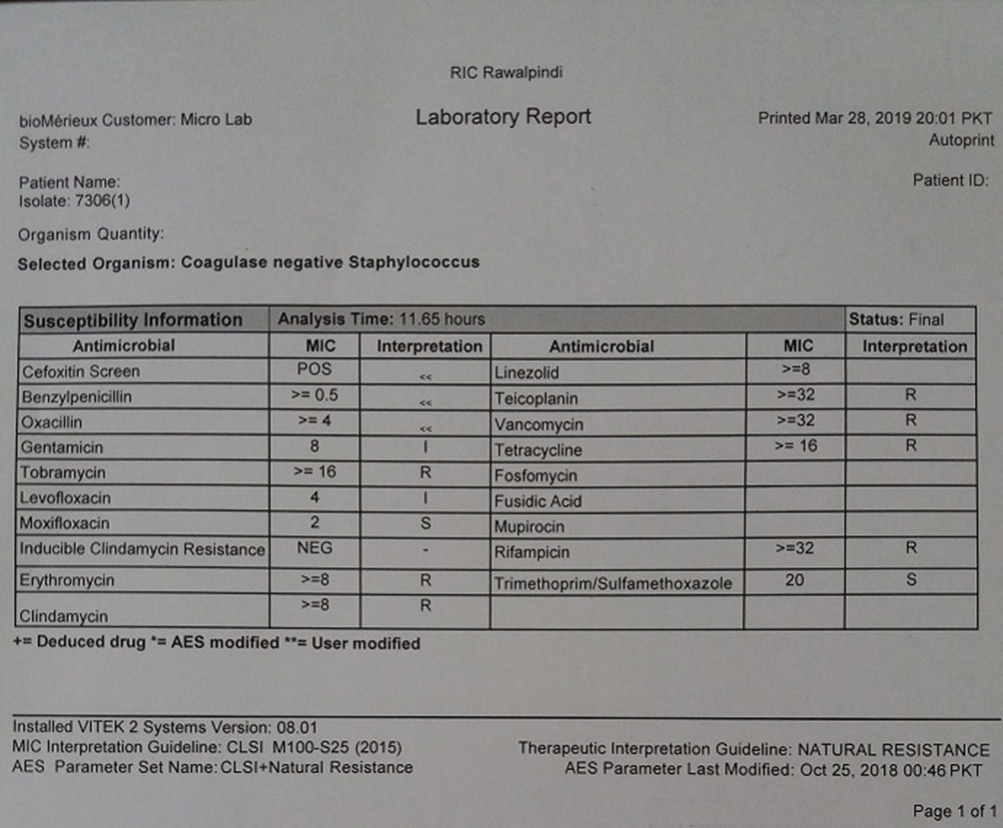
Susceptibility results of *K. kristinae *.

Species identification was performed using Vitek 2, bioMerieux, France automated blood identification system (GP Card REF 21 342). Since there are no recommended guidelines of antibiotic susceptibility for *Kocuria *, it was carried out in Vitek-2 using AST Card -P 580 bioMerieux, France as for as *Coagulase-negative Staphylococcus* [13]. All three culture isolates were identified as *K. kristinae* (Fig. 2) to a probability level of 88 to 95 %. These were susceptible to moxifloxacin, trimethoprim/sulphamethoxazole, intermediately susceptible to levofloxacin, gentamycin, and resistant to erythromycin, clindamycin, tobramycin, vancomycin, teicoplanin, tetracycline, rifampicin as per CLSI break points (Fig. 3). Since cefoxitin screen was positive by Vitek and Kirby–Bauer both penicillins and cephalosporins were reported as resistant. The same was also confirmed by the modified Kirby–Bauer disc-diffusion method [13]. As there are no current guidelines for the treatment, based upon the susceptibility results i.v vancomycin and i.v amikacin initially started empirically were stopped and replaced with i.v moxifloxacin 400 mg twice daily and i.v gentamycin 80 mg 8 hourly [14]. Daptomycin an alternate antibiotic to vancomycin for native valve endocarditis was not available as a treatment option. The organism was also tested to be resistant to clindamycin.

Patient became afebrile after 24 h on new regimen of intravenous antibiotics. Her WBC counts and CRP gradually decreased and were within normal limits beside negative blood cultures before discharge from hospital after 2 weeks of inpatient therapy. She was advised to follow up 2 weeks after being discharged.

## Discussion

Right-sided endocarditis is relatively rare and commonly affects tricuspid valve. Endocarditis due to infection of pulmonary valve is even more rarely reported than tricuspid valve endocarditis and is responsible for about 2 % of hospital admissions for endocarditis [15]. Right-sided endocarditis is less frequent than left-sided endocarditis probably due to different haemodynamic pressure gradients across the valves, frequencies of underlying valvular abnormalities and lower blood oxygen content in the right side of heart [16, 17]. It has better prognosis with lower mortality. Often, it is managed conservatively with antibiotics and surgery is required in the case of secondary heart failure, lung involvement and septic embolism [15].

All three sets of blood cultures of our patient taken 12 hourly apart as per guidelines yielded growth of *K. kristinae* [14]. It is a Gram-positive coccus, catalase-positive, coagulase-negative, DNAase-negative and is a facultative anaerobe. Major criteria for the conventional discrimination between *Micrococci* and *Staphylococci* are the sensitivity of *Kocuria* to bacitracin and lysozyme (while *Staphylococci* are resistant to both) and the resistance of *Kocuria* to nitrofurantoin/furazolidone and lysostaphin (*Staphylococci* are susceptible to the latter, although they may express resistance to the former) [6]. Our isolates also showed resistance to nitrofurantoin but sensitivity to bacitracin.

Previously *Kocuria* was classified under the genus *Micrococcus* and regarded as a harmless normal skin microbiota. However, it has now been reclassified under the new genus *Kocuria* [18]. Species-level identification requires an automated identification system and other molecular methods [7]. Genomic methods, as 16S RNA gene sequence and matrix-assisted laser desorption/ionization time-of-flight mass spectrometry (MALDI-TOF-MS) are desirable for correct identification of *Coagulase-negative Staphylococcus,* which presents a large phenotypic variation [7]. This kind of approach is equally useful to confirm *Kocuria* species [19].

The majority of studies included in a review used biochemical methods, especially Vitek-2 system bioMerieux, France, for identification of *K. kristinae *. Although, misidentification of *Coagulase-negative Staphylococcus* as *Kocuria* spp. with the Vitek system has been reported with an early version of this phenotype-based system, the enhanced versions in the form of Vitek-2 are not prone to error [5]. Boudewijns *et al*. have reported that the recently introduced Vitek-2 Gram-positive GP identification card and database by bioMerieux, France, allows identification of additional taxa including *K. kristinae* [20]. Many recent studies including ours, correctly identified *Kocuria* spp. using the Vitek-2 Gram-positive identification card, due to the recently introduced larger database that allows the identification of additional taxa [4, 21]. Although, the genome analysis through molecular methods is desirable, due to economic and technical limitations, its use was not possible in our set up.

*Kocuria* are widely distributed in nature and are found frequently as a part of normal skin and oral microbiota in humans and other mammals [6]. The genus has more than 18 species. Among these, only five are known to be opportunistic pathogens [7]. There are only a few reports of *K. kristinae* infections often isolated in patients with malignancies or other immunosuppressed states [13]. *K. kristinae* along with *Leuconostoc mesenteroides* are among the micro-organisms associated under carious teeth [8]. In our patient with congenital heart disease from low socioeconomic class with poor dental hygiene and probably the use of over the counter antibiotics by GPs for prolonged and undiagnosed fever probably lead to *K. kristinae* endocarditis. Moreover, the possibility of healthcare-associated infection due to frequent injections/cannulations could not be ruled out. Only five patients have been reported in the literature with IE caused by *K. kristinae* until 2016 [12]. Although the policy of individual diagnostic laboratory to report the the organism may vary, it is important that in practice, it may not be misidentified as *Coagulase-negative Staphylococcus* as was initially reported in our patient [22].

There are no internationally accepted guidelines for antibiotic treatment of IE caused by *K. kristinae* infection [6]. We report a vancomycin-resistant *K. kristinae* isolate, which is an unusual finding that needs to be followed in further studies.

The majority of patients were treated with vancomycin as monotherapy or in combination with one or two other antibiotics [22, 23]. However, in our case it was sensitive to moxifloxacin and gentamycin but resistant to vancomycin and cephalosporins. CLSI has a recommended MIC of vancomycin less than 4 and >32 ug ml^−1^ for susceptibility and resistance, respectively, for all *Coagulase-negative Staphylococcus* [13]. Our *K. kristinae* isolates had MIC*>*32 ug ml^−1^, which was also confirmed by E strip and were therefore reported to be resistant to vancomycin. Vancomycin resistance in *Kocuria* observed in our study is unusual. Further studies are required to investigate vancomycin resistance in *Kocuria *. In the absence of specific MIC, inhibiting zone diameter and break points on agar media of *K. kristinae,* the sensitivity and resistance results cited in the literature are based on *Coagulase-negative Staphylococcus* interpretive values [6, 13].

Vancomycin and cephalosporin-resistant isolates have already been reported in a case study from Delhi, India [24]. Antibiotic resistance in the organism has also been reported from Egypt, where it was found to be sensitive to cefoxitin, gentamycin, amikacin, ciprofloxacin, levofloxacin and linezolid but resistant to vancomycin, teicoplanin, rifampicin, amoxicillin/clavulanate and clindamycin [4]. The antibiogram observed in our study reflects the resistance of the organism to different antibiotics in healthcare facilities and possible risk of acquisition as a healthcare-associated infection. Antibiotic resistance to a large number of antibiotics is an emerging threat to treat such infections.

Pulmonary valve endocarditis even in the presence of structural heart disease is very rare. A careful history, clinical examination in a patient with suspected endocarditis must be confirmed by echocardiography and supported by positive blood cultures to confirm the etiology. Unusual micro-organisms such as *K. kristinae* should be kept in mind as a cause of IE especially in patients with congenital heart disease.

### Limitations of study

Lack of sequencing to confirm the identity of the isolate due to economic constraints and technical limitations.
